# Size-Dependent Metabolic Reprogramming in A549 Cells Induced by Mesoporous Silica Nanoparticles: Insights from Subcellular Targeting

**DOI:** 10.3390/metabo16080559

**Published:** 2026-08-07

**Authors:** Jing Li, Hui Xu

**Affiliations:** 1 Key Laboratory of Modeling, Simulation and Control of Complex Ecological Model in Dabie Mountains of Anhui Higher Eduction Institutes, School of Physics and Astronomy, Anqing Normal University, Anqing 246133, China; 2 Key Laboratory of Drug Metabolism and Pharmacokinetics, State Key Laboratory of Natural Medicines, China Pharmaceutical University, Nanjing 210009, China; xuhui@csu.edu.cn; 3 Laborator of Nano-Biology Technology, Institute of Super-Microstructure and Ultrafast Process in Advanced Materials, School of Physics, Central South University, Changsha 410083, China

**Keywords:** mesoporous silica nanoparticles, metabolomics, size-dependent toxicity, mitochondria, inflammation, metabolic reprogramming

## Abstract

**Background/Objectives**: Mesoporous silica nanoparticles (MSNs) are widely investigated as nanocarriers for drug delivery, gene transfer, and bioimaging. However, the mechanisms underlying their size-dependent cytotoxicity at the metabolic level remain incompletely understood. This study aimed to determine whether different-sized MSNs induce distinct patterns of subcellular injury and metabolic reprogramming in lung epithelial cells. **Methods**: A549 cells were exposed to 80 nm and 600 nm MSNs at 50 and 200 μg/mL for 24 h. Ultrastructural changes were examined by transmission electron microscopy (TEM). Intracellular reactive oxygen species (ROS) and Ca^2+^ were measured by 2′,7′-dichlorodihydrofluorescein diacetate (DCFH-DA) and Fluo-4 AM fluorescence, respectively. Inflammatory gene expression (IL1B, IL6, TNFA, HIF1A) was quantified by reverse transcription quantitative polymerase chain reaction (RT-qPCR). Untargeted metabolomics were performed using combined gas chromatography–mass spectrometry (GC-MS) and liquid chromatography–mass spectrometry (LC-MS) platforms, followed by principal component analysis (PCA), partial least squares discriminant analysis (PLS-DA), and MetaboAnalyst-based pathway enrichment. **Results**: TEM revealed distinct size-dependent subcellular distributions: 80 nm MSNs were predominantly associated with mitochondrial abnormalities, including cristae disruption, swelling, and mitophagy-like features, whereas 600 nm MSNs accumulated in endocytic vesicles with membrane disruption. Metabolomic profiling showed that 80 nm MSNs were associated with TCA cycle blockade—characterized by the accumulation of early intermediates (citrate, oxaloacetate) and the depletion of distal intermediates (fumarate, malate)—with compensatory glycolytic activation (increased glyceraldehyde-3-phosphate and pyruvate) and reduced deoxynucleotide pools (dCDP, dUMP). By contrast, 600 nm MSNs triggered broad nucleotide triphosphate accumulation (ATP, CTP, dGTP, dCTP), amino acid depletion, and robust inflammatory activation, including a ~136-fold increase in IL1B expression and HIF1A transcriptional upregulation. PCA and PLS-DA confirmed distinct size-dependent metabolic phenotypes. **Conclusions**: MSN size strongly influences subcellular targeting—80 nm particles were predominantly associated with mitochondrial injury while 600 nm particles disrupted endocytic vesicles—driving qualitatively distinct patterns of metabolic reprogramming and inflammatory signaling. These findings establish a correlative mechanistic framework linking particle size to organelle-specific injury and provide candidate metabolic markers for nanotoxicological evaluation.

## 1. Introduction

Mesoporous silica nanoparticles have attracted considerable attention in biomedical research because of their ordered porous structure, large surface area, tunable pore size, and amenability to surface modification. These features make MSNs suitable for controlled drug release, gene delivery, molecular imaging, and theranostic applications [1,2,3]. Alongside their expanding use, however, concerns have increased regarding their potential adverse effects, especially following occupational, environmental, or biomedical exposure [4,5,6]. Accumulating evidence indicates that the biological identity and toxicity of nanomaterials are strongly influenced by physicochemical parameters, such as size, morphology, surface charge, porosity, and surface chemistry [7,8,9,10]. Among these properties, particle size is particularly important because it affects cellular uptake, intracellular trafficking, organelle interaction, clearance, and inflammatory potential [11,12,13].

The respiratory tract is a major route of exposure to airborne nanomaterials [14,15,16]. Inhaled silica-based nanoparticles may deposit in the alveolar region, where they interact with epithelial cells and resident immune cells, leading to oxidative stress, mitochondrial injury, inflammatory activation, and tissue remodeling [17,18,19]. A549 cells, derived from human lung adenocarcinoma, are widely used as an in vitro model for pulmonary epithelial responses to nanomaterials. Although this model does not fully reproduce the behavior of normal lung epithelium, it provides a reproducible platform for examining nanoparticle uptake, cytotoxicity, oxidative stress, and metabolic changes. Previous studies have shown that silica nanoparticles can induce ROS accumulation, mitochondrial dysfunction, membrane damage, and inflammatory cytokine release in pulmonary and other cell types [17,20]. Nevertheless, the relationship between MSN size, subcellular targeting, and metabolic reprogramming remains poorly defined.

In the present study, we selected two MSN sizes (80 nm and 600 nm) to represent distinct size regimes relevant to cellular uptake and intracellular trafficking. Particles in the sub-100 nm range are known to access multiple subcellular compartments and may interact directly with organelles, such as mitochondria, whereas larger particles (hundreds of nanometers) are generally confined to endocytic vesicles and may exert effects primarily through vesicular and membrane stress [11,12,13]. This size contrast therefore provides an opportunity to examine how particle size governs subcellular targeting and the ensuing metabolic consequences.

Metabolomics offer a sensitive means of capturing the downstream biochemical consequences of nanomaterial exposure [21]. Because metabolites are close to the functional phenotype, metabolomic profiling can reveal changes in energy metabolism, redox balance, amino acid turnover, nucleotide synthesis, and stress adaptation that may not be apparent from conventional cytotoxicity assays alone [21,22,23]. Both GC-MS and LC-MS platforms have been increasingly used in nanotoxicology to identify metabolic fingerprints of exposure and to explore mechanisms of toxicity. However, few studies have integrated metabolomics with ultrastructural evidence to determine how different-sized MSNs perturb specific subcellular compartments and thereby drive distinct metabolic outcomes.

In the present study, we investigated the size-dependent effects of 80 nm and 600 nm MSNs in A549 cells. We combined cell viability assays, ROS and intracellular Ca^2+^ measurements, RT-qPCR analysis of inflammatory markers, TEM observation, and untargeted GC-MS/LC-MS metabolomics. This integrated approach was designed to address three questions: whether different-sized MSNs are associated with distinct organelles; whether metabolic profiles can explain size-dependent toxicity; and how metabolic perturbation is linked to oxidative stress and inflammatory signaling. By establishing a particle size–subcellular targeting–metabolic reprogramming framework, this study provides a correlative mechanistic insight into MSN-induced toxicity and informs the design of safer silica-based nanomaterials.

## 2. Materials and Methods

### 2.1. Synthesis and Characterization of Mesoporous Silica Nanoparticles

For mesoporous silica nanoparticles of different sizes, their synthesis and characterization were performed as previously reported [24]. Briefly, n-octadecyltrimethoxysilane (C18TMS, 95%) and tetraethyl orthosilicate (TEOS, 98%) were mixed in 50 mL ethanol, sonicated for 10 min, transferred to ethanol/ammonia/deionized water solution, and then stirred at room temperature for 2 h. C18TMS particles of different sizes were collected by filtration, washed with ethanol and deionized water, and dried at room temperature. Finally, samples were calcined at 550 °C for 6 h to remove the organic template, yielding pure silica nanoparticles. The morphology and structure of mesoporous silica nanoparticles were observed using Hitachi S-4800 scanning electron microscopy (SEM). The particles were spherical, with a primary diameter of approximately 80 nm and 600 nm. Hydrodynamic diameters measured by dynamic light scattering (DLS) in complete culture medium were 236.7 ± 19.0 nm and 1237.8 ± 76.2 nm for the 80 nm and 600 nm particles, respectively. Zeta potential values were −9.05 ± 1.47 mV and −10.85 ± 1.94 mV for the 80 nm and 600 nm particles, respectively. Representative SEM images are provided in Figure S1.

### 2.2. Cell Culture

Human non-small cell lung cancer (A549) cells were obtained from the American Type Culture Collection and cultured in RPMI-1640 medium (Gibco; Thermo Fisher Scientific, Waltham, MA, USA), supplemented with 10% fetal bovine serum (FBS; Gibco), 100 U/mL penicillin, and 0.1 mg/mL streptomycin (Gibco, 100×, 10,000 U/mL penicillin, 10 mg/mL streptomycin). Cells were maintained at 37 °C in a humidified atmosphere containing 5% CO_2_. For metabolomic analysis, cells were seeded at a density of 2 × 10^5^ cells per well in 6-well plates and cultured for 24 h to reach approximately 90% confluence. Cells were then treated for 24 h with 80 nm or 600 nm MSNs dispersed in the culture medium. The experimental groups were the control, 50 μg/mL 80 nm MSN, 200 μg/mL 80 nm MSN, 50 μg/mL 600 nm MSN, and 200 μg/mL 600 nm MSN groups, with six biological replicates for each group. After treatment, the medium was removed and cells were rinsed twice with cold phosphate-buffered saline.

### 2.3. MTT Assay

Cell viability was assessed using the MTT assay. A549 cells were seeded in 96-well plates and treated with MSNs at 0, 50, 100, 200, 500, and 1000 μg/mL for 24 h. After exposure, the medium was removed and the cells were washed with PBS; then, 20 μL of MTT solution (Sigma-Aldrich, St. Louis, MO, USA) was added to each well and the cells were incubated at 37 °C for 4 h. The medium was removed, and formazan crystals were dissolved in 150 μL of dimethyl sulfoxide. Absorbance was measured at 570 nm with a reference wavelength of 695 nm using a microplate reader.

### 2.4. Measurement of Intracellular ROS

Intracellular ROS production was determined using DCFH-DA (Beyotime, Shanghai, China). DCFH-DA (10 μM) was added for the final 4 h of the 24 h MSN exposure period (co-incubation), and the cells were maintained at 37 °C in the dark. This extended co-incubation was chosen to capture cumulative ROS production over a prolonged exposure window instead of a snapshot of instantaneous ROS levels, as nanoparticles may induce sustained oxidative stress over time. Cells were then collected, and fluorescence intensity was measured at excitation and emission wavelengths of 488 and 535 nm, respectively.

### 2.5. Measurement of Intracellular Ca^2+^

A549 cells were treated with 80 nm or 600 nm MSNs at 50 or 200 μg/mL for 24 h. After washing with PBS, cells were loaded with Fluo-4 AM working solution for 30 min at 37 °C. The cells were washed again and incubated for an additional 30 min to allow complete intracellular de-esterification of the probe. Fluorescence was measured at excitation and emission wavelengths of 488 and 516 nm, respectively.

### 2.6. Transmission Electron Microscopy

Cells were treated with 80 nm or 600 nm MSNs at 50 or 200 μg/mL for 24 h. After treatment, cells were washed with PBS and then harvested and fixed in 2.5% glutaraldehyde for 1 h. Samples were post-fixed with 1% osmium tetroxide, dehydrated through graded ethanol, embedded in epoxy resin, and then sectioned. Ultrathin sections were stained with uranyl acetate and lead citrate and observed using a Hitachi H-7650 transmission electron microscope.

### 2.7. RT-qPCR

Total RNA was isolated using TRI Reagent (Sigma-Aldrich). RNA concentration and purity were determined with a NanoDrop ND-1000 spectrophotometer. RNA integrity was assessed using the A260/A280 ratio (all samples: 1.8–2.1). One microgram of total RNA was reverse-transcribed into cDNA using a commercial reverse transcription kit (Takara Bio, Kusatsu, Japan). Quantitative PCR was performed using SYBR Green detection on an ABI ViiA 7 Real-Time PCR System. ACTB (β-actin) and GAPDH were used as internal reference genes, and the geometric mean of both genes was used for normalization. Raw Ct values and ΔCt analysis supporting reference gene stability are provided in Supplementary Table S6. Primer sequences are provided in Supplementary Table S3.

### 2.8. Metabolomic Analysis

#### 2.8.1. GC-MS Analysis

For GC-MS analysis, metabolites were extracted by adding 200 μL water and 800 μL methanol containing [1,2-^13^C_2_] myristic acid as an internal standard. Samples were centrifuged at 20,000× *g* for 10 min, and 120 μL of the supernatant was transferred to GC vials and evaporated to dryness using an SPD2010-230 SpeedVac Concentrator (Thermo Savant, Holbrook, AZ, USA). Methoxyamine (30 μL) in pyridine (10 mg/μL) was added to the dried GC vial and shaken to dissolve the metabolites for 5 min. The methoximation reaction proceeded for 16 h at room temperature. Then, 30 μL of N-methyl-N-(trimethylsilyl) trifluoroacetamide (MSTFA) with 1% TMCS was added to the trimethylsilylation reaction for 1 h. Finally, 30 μL of heptane, including methyl myristate (30 μg/mL), was added to each solution, and the solution was mixed by vortexing for another 1 min before analysis. Then, 0.5 μL aliquots were injected using Agilent 7683 series autoinjector (Agilent Technologies, Santa Clara, CA, USA) onto a 10 m × 0.18 mm i.d. fused silica capillary column with 0.18 μm DB-5MS stationary phase (J&W Scientific, Folsom, CA, USA). Mass spectra were acquired from *m*/*z* 50 to 680 at 30 spectra/second after a 175 s solvent delay. Column temperature was initially held at 80 °C for 3 min, then ramped to 300 °C and held for 5 min. Automated peak detection was performed using Chroma TOFTM software (Leco, version 3.25). Endogenous metabolites were identified by comparing mass spectra and retention indices with the reference standards or available libraries: National Institute of Standards and Technology (NIST) library 2.0 (2008) and Wiley 9 (Wiley–VCH Verlag GmbH & Co. KGaA, Weinheim, Germany). Each peak area was normalized by an internal standard (IS).

#### 2.8.2. LC-MS Analysis

For LC-MS analysis, we added 200 μL of water and 600 μL of methanol solvent containing the internal standard (IS) (^13^C-glutamine; methanol:water ratio = 4:1 *v*/*v*) to the 6-well plate to precipitate proteins and extract metabolites. After centrifugation at 12,000× *g* for 10 min, 400 μL clear supernatant was transferred to new Eppendorf tubes and evaporated to dryness. Residues were reconstituted with 100 μL ultrapure water, and 20 μL supernatant was analyzed by LCMS-Q-TOF. HPLC separation was performed on a Waters Amide XBridge HPLC column (4.6 × 100 mm, 3.5 μm, Waters, Milford, MA, USA) and maintained at 30 °C. The mobile phase consisted of solvent A (aqueous solution containing 5 mM ammonium acetate, pH adjusted to 9 with ammonia, 5% acetonitrile) and solvent B (acetonitrile). Gradient elution was used with the following program: 0–3 min 85% B, 3–6 min 85–30% B, 6–15 min 30–2% B, 15–18 min 2% B, 18–19 min 2–85% B, and 19–26 min 85% B. The flow rate was 0.4 mL/min. Mass spectrometry was performed in negative electrospray ionization (ESI) mode. The ion mode scan range and product ion scan range were 50–1000 and 50–900 *m*/*z*, respectively. The ion source gas 1 (GAS 1), ion source gas 2 (GAS 2), and curtain gas were set at 33, 33, and 25 psi, respectively. Other MS parameters included: ion spray voltage -4500 V; turbo spray temperature 500 °C; declustering potential (DP) 93 V; TOF MS scan collision energy (CE) −10 V; and accurate mass calibrated via calibration delivery system (CDS). Each of the 6 samples was automatically calibrated using the reference masses.

#### 2.8.3. Metabolomic Data Processing

For both GC-MS and LC-MS datasets, metabolite features were filtered to retain those present in ≥80% of samples within at least one experimental group. Missing values were imputed using the k-nearest neighbors (kNN) algorithm (k = 5). Peak areas were normalized to the internal standard before statistical analysis. Differentially altered metabolites were identified using a combined criterion: fold-change > 1.2 or <0.83 (|log2(FC)| > 0.26); VIP score > 1.0 from PLS-DA; and raw *p*-value < 0.05 (Student’s *t*-test or one-way ANOVA). The Benjamini–Hochberg false discovery rate (FDR) correction was applied, and metabolites with FDR < 0.25 were considered significantly altered—a threshold commonly used in untargeted metabolomic studies where strict FDR control may exclude biologically relevant features in small-to-moderate sample sizes.

### 2.9. Statistical Analysis

Data are presented as mean ± SD from at least three independent experiments. Statistical analysis was performed using GraphPad Prism 8.0. Differences between groups were assessed by one-way ANOVA followed by Dunnett’s post hoc test for comparisons against the control group. A value of *p* < 0.05 was considered statistically significant. Principal component analysis (PCA) and partial least squares discriminant analysis (PLS-DA) were performed using SIMCA 14.1. Metabolomic pathway enrichment analysis was performed using MetaboAnalyst 6.0 (https://www.metaboanalyst.ca, accessed on 2 August 2026) with the Homo sapiens pathway library (KEGG, 2025 release).

## 3. Results

### 3.1. Size-Dependent Subcellular Distribution and Organelle Injury

**Figure 1:**
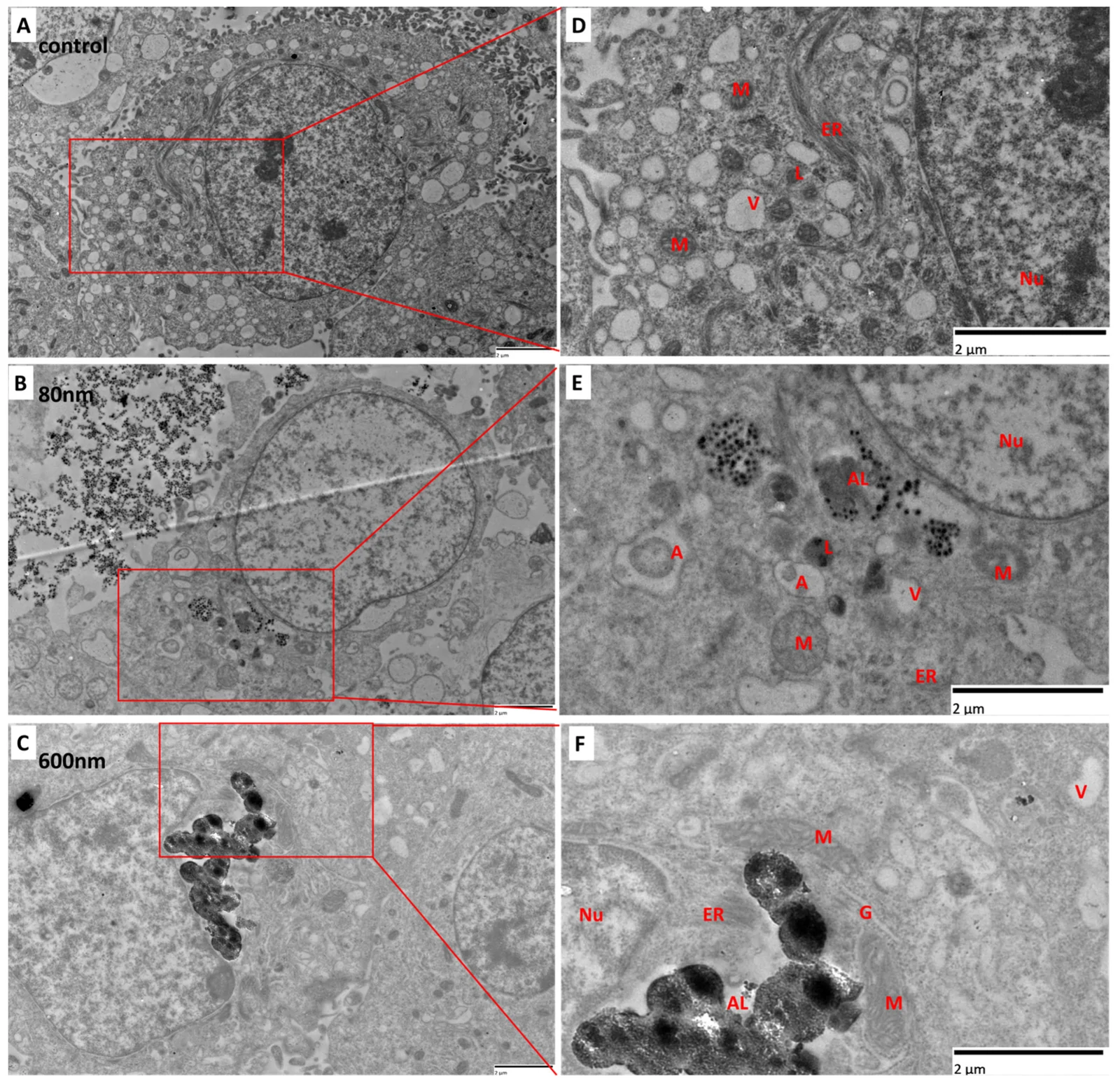
Cellular uptake of MSNs and the following ultrastructural alterations by TEM analysis. (**A**,**D**) Control group, (**B**,**E**) 80 nm group, and (**C**,**F**) 600 nm group. A549 cells were exposed to 200 μg/mL MSNs for 24 h. L, lysosome; A, autophagosome; M, mitochondria; ER, endoplasmic reticulum; Nu, nucleus; G, Golgi apparatus; V, vesicle.

Both 80 nm and 600 nm MSNs were internalized by A549 cells (Figure 1). TEM analysis showed that particles were mainly distributed in the cytoplasm and were not observed inside the nucleus. However, 600 nm MSNs occupied larger intracellular spaces and compressed adjacent nuclear structures, resulting in slight nuclear deformation (Figure 1C). The intracellular behavior of the two particle sizes differed substantially. The 80 nm MSNs were mainly enclosed within membrane-bound vesicles with relatively intact boundaries (Figure 1B), whereas 600 nm MSNs were associated with disrupted vesicular membranes and less clearly defined vesicle structures.

Further ultrastructural examination showed that cells exposed to 80 nm MSNs frequently exhibited mitochondrial abnormalities, including swelling, rounded morphology, and loss or rupture of cristae and features of mitophagy (Figure 1E). By contrast, mitochondria in the 600 nm MSN group were less affected morphologically, whereas vesicular disruption, reduced cytoplasmic space and nuclear compression were more pronounced. These observations suggest that smaller MSNs were predominantly associated with mitochondrial injury, while larger MSNs appeared to primarily disturb endocytic vesicles and intracellular membrane organization (Figure 1F). These TEM findings are descriptive in nature and were not subjected to quantitative morphometric analysis, which represents a limitation of the present study.

### 3.2. ROS Production, Calcium Imbalance, and Inflammatory Activation

**Figure 2:**
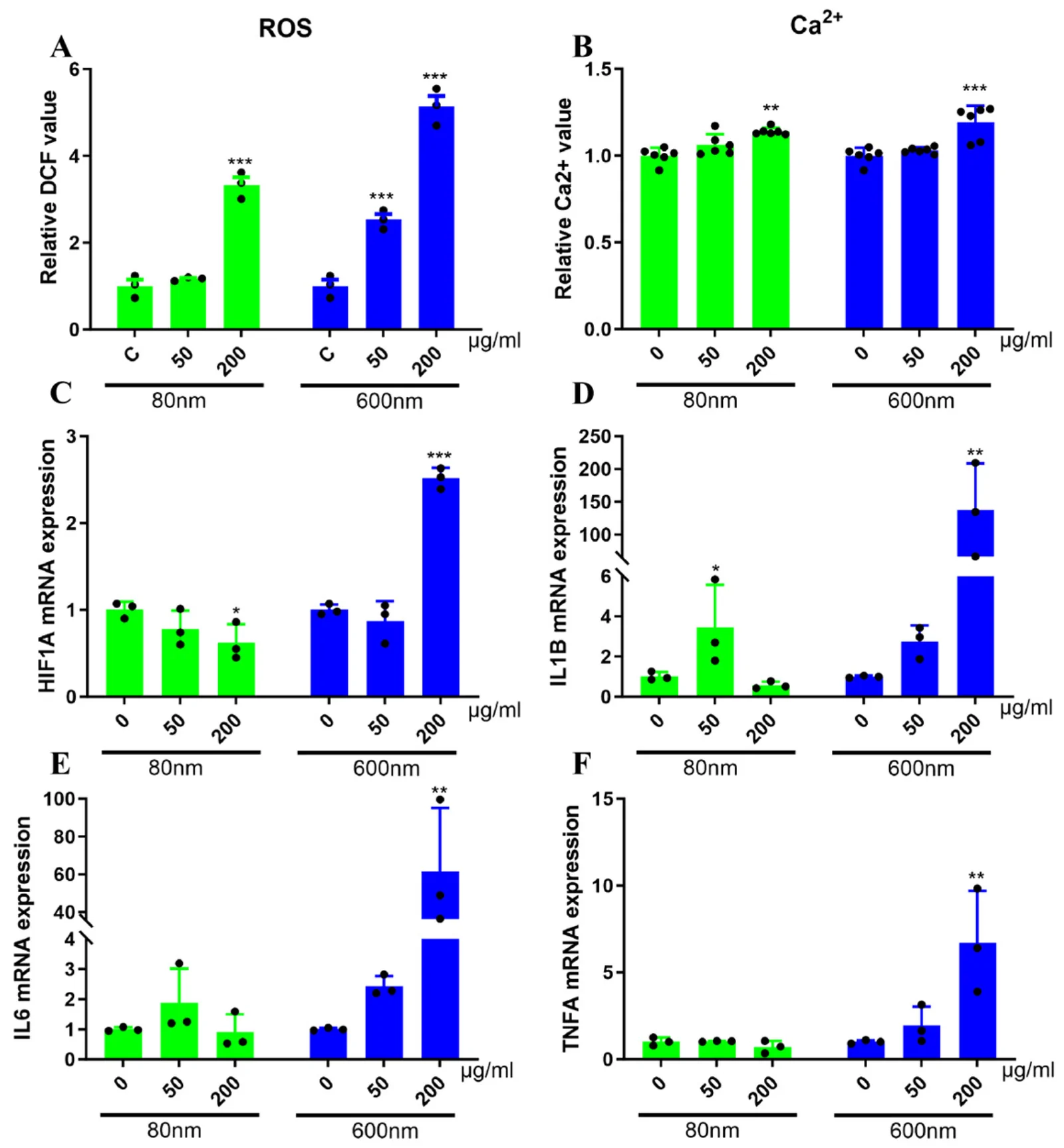
ROS production, calcium imbalance, and inflammatory activation. (**A**) Intracellular ROS levels after treatment with MSNs for 24 h (DCFH-DA added for the final 4 h), measured by DCF fluorescence. (**B**) Intracellular Ca^2+^ levels after treatment with MSNs for 24 h, measured by Fluo-4 AM. (**C**–**F**) Relative mRNA levels of HIF1A (**C**), IL6 (**D**), TNFA (**E**), and IL1B (**F**), determined by RT-qPCR after 24 h MSN exposure. Data are expressed as mean ± SD from three independent experiments. * *p* < 0.05, ** *p* < 0.01, and *** *p* < 0.001 compared with the control group (one-way ANOVA with Dunnett’s post hoc test). Individual data points (black dots) represent independent biological replicates.

Cell viability, as assessed using the MTT assay, showed dose-dependent cytotoxicity for both particle sizes (Supplementary Figure S2). The 600 nm MSNs exhibited greater cytotoxicity than the 80 nm MSNs at equivalent mass concentrations, with viability decreasing to ~75% at 50 μg/mL and ~50% at 200 μg/mL for 600 nm particles, compared with >80% and ~65% for 80 nm particles. Because mitochondrial injury and vesicular membrane damage can both promote oxidative stress, ROS levels were measured using DCF fluorescence. MSN exposure increased intracellular ROS in a dose-dependent manner. The 600 nm MSNs induced a more pronounced increase in ROS than the 80 nm MSNs, particularly at the higher concentration (Figure 2A). Intracellular Ca^2+^ levels also increased with the MSN dose, although the difference between particle sizes was less evident (Figure 2B).

Inflammatory gene expression was then analyzed by RT-qPCR. IL6, IL1B, and TNFA transcripts were upregulated after MSN treatment, with stronger induction observed in cells exposed to 600 nm MSNs and at higher doses. In the 600 nm high-dose group, IL6, TNFA, and IL1B increased by 61.6-, 6.7-, and approximately 136-fold (Figure 2D–F), respectively. In contrast, 80 nm MSNs induced a more limited inflammatory response, with significant IL6 upregulation mainly at the lower dose. HIF1A expression was significantly increased in the 600 nm high-dose group but not in the low-dose group. In the 80 nm group, HIF1A was downregulated at the higher dose and showed no significant change at the lower dose (Figure 2C). These results indicate that larger MSNs provoke stronger inflammatory responses and HIF1A transcriptional upregulation.

### 3.3. Global Metabolomic Profiling

**Figure 3:**
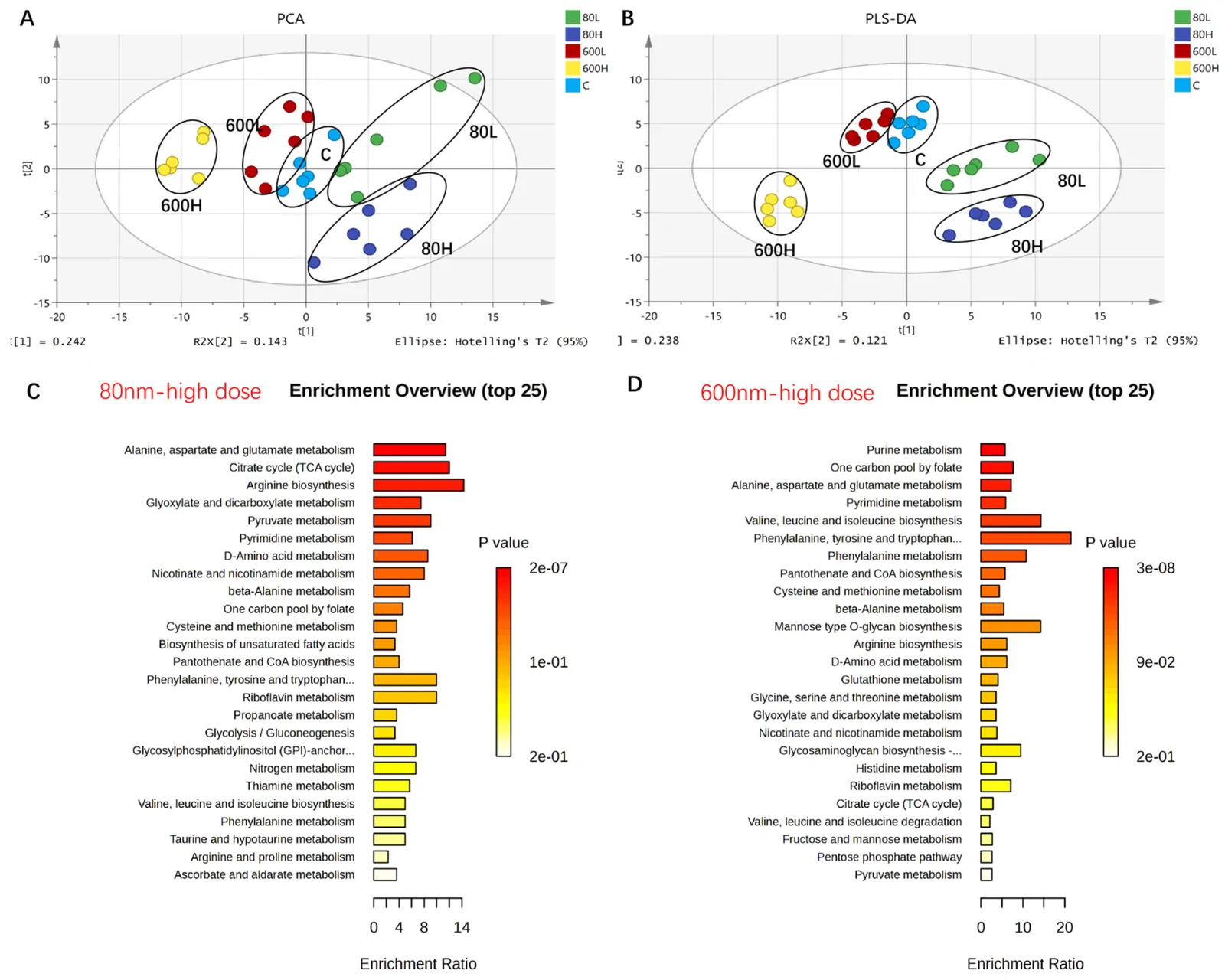
Global metabolomic profiling. (**A**) PCA scores plot and (**B**) PLS-DA scores plot showing metabolic discrimination between control, 80 nm (low and high dose), and 600 nm (low and high dose) MSN-treated groups. Each point represents an individual biological replicate. Ellipses represent the 95% confidence region. (**C**) Metabolic pathway enrichment and topology analysis for the 80 nm high-dose group versus control. (**D**) Metabolic pathway enrichment and topology analysis for the 600 nm high-dose group versus control.

Untargeted metabolomics were used to evaluate the metabolic consequences of MSN exposure. PCA is an unsupervised method that identifies the directions of maximum variance without using group labels, whereas PLS-DA is a supervised method that maximizes separation between predefined groups. The PLS-DA model achieved Q^2^ = 0.803, R^2^X = 0.549 and R^2^Y = 0.928, confirming that the observed group separation was statistically significant and not due to overfitting. Both PCA and PLS-DA demonstrated clear separation between control and MSN-treated groups, indicating substantial metabolic remodeling. Samples within each group clustered closely, supporting the reproducibility of the metabolomic data. The 80 nm and 600 nm groups were positioned on opposite sides of the control group in multivariate score plots, suggesting that the two particle sizes induced distinct metabolic phenotypes. High-dose groups were located farther from the controls, consistent with dose-dependent metabolic disturbance (Figure 3A,B).

Using the combined GC-MS and LC-MS platforms, pathway enrichment analysis was performed on differentially altered metabolites for each MSN-treated group versus its corresponding control group. In the 80 nm high-dose group, altered pathways included alanine, aspartate, and glutamate metabolism; the TCA cycle; arginine biosynthesis; pyruvate metabolism; pyrimidine metabolism; and nicotinate and nicotinamide metabolism (Figure 3C). In the 600 nm high-dose group, the main affected pathways included pyrimidine and purine metabolism; one carbon pool by folate; alanine, aspartate, and glutamate metabolism; and essential amino acid biosynthesis (Figure 3D).

### 3.4. Distinct Energy Metabolic Responses to 80 nm and 600 nm MSNs

Energy metabolism was markedly affected by MSN exposure, but the direction and pattern of alteration depended on particle size. In the 80 nm low-dose group, citrate (FC = 1.37), oxaloacetate (FC = 1.37), cis-aconitate (FC = 1.37), N-acetylornithine (FC = 8.89), and pyruvate (FC = 1.60) were significantly increased, whereas fumarate (FC = 0.68) and malate (FC = 0.69) were significantly decreased (Supplementary Table S1, Figure S3). In the 80 nm high-dose group, citrate (FC = 1.89), oxaloacetate (FC = 2.02), cis-aconitate (FC = 1.97), glyceraldehyde 3-phosphate (FC = 1.70), pyruvate (FC = 1.97), and phosphoenolpyruvate (FC = 1.45) were significantly increased (Supplementary Table S1, Figure S3). GC-MS analysis corroborated these findings, showing that at the 200 μg/mL dose there was citric acid accumulation (FC = 3.20, *p* < 0.05), with concurrent decreases in fumaric acid (FC = 0.80), malic acid (FC = 0.76), and succinic acid (FC = 0.78), consistent with TCA cycle blockade (Supplementary Table S3). The accumulation of early TCA cycle intermediates (citrate, cis-aconitate, oxaloacetate), together with the depletion of downstream intermediates (fumarate, malate, succinate) and elevation of glycolytic intermediates (glyceraldehyde-3-phosphate, pyruvate), is consistent with mitochondrial impairment and compensatory glycolytic activation.

In contrast, 600 nm MSNs elicited a distinct metabolic response. In the 600 nm low-dose group, the primary alterations involved amino acid depletion, where isoleucine (FC = 0.73), methionine (FC = 0.74), tryptophan (FC = 0.76), tyrosine (FC = 0.77), asparagine (FC = 0.74), and glutamine (FC = 0.73) were significantly decreased, while hypoxanthine was reduced (FC = 0.82) (Supplementary Table S2). GC-MS analysis confirmed amino acid depletion at the 50 μg/mL dose, where aspartic acid (FC = 0.78), glutamine (FC = 0.76), ornithine (FC = 0.74), and serine (FC = 0.82) were decreased, while citric acid (FC = 1.41) and 2-ketoglutaric acid (FC = 1.49) were elevated (Supplementary Tables S2 and S4). In the 600 nm high-dose group, energy metabolism changes were less pronounced than in the 80 nm group: glyceraldehyde-3-phosphate was increased (FC = 1.98), whereas TCA cycle intermediates were not significantly altered in LC-MS. GC-MS analysis at the 200 μg/mL dose confirmed this pattern, as citric acid was elevated (FC = 1.81), but fumaric acid (FC = 0.84) and malic acid (FC = 0.88) showed no significant changes, while amino acid depletion was prominent—aspartic acid (FC = 0.48), glutamine (FC = 0.51), and ornithine (FC = 0.70) were substantially decreased. These results indicate that 600 nm MSNs primarily induced amino acid depletion and metabolic stress rather than direct TCA cycle blockade, consistent with a mechanism involving vesicular stress and inflammatory activation rather than primary mitochondrial impairment.

### 3.5. Dysregulation of Nucleotide Metabolism

Purine and pyrimidine metabolism showed prominent size- and dose-dependent alterations. In the 80 nm low-dose group, uracil (FC = 1.46), cytidine monophosphate (FC = 1.24), and NADPH (FC = 0.67) were altered, while dCMP (FC = 0.75) and dUMP (FC = 0.76) were decreased (Supplementary Table S1, Figure S4). In the 80 nm high-dose group, uracil (FC = 1.84), inosine (FC = 1.43), cytidine monophosphate (FC = 1.48), and cyclic AMP (FC = 1.32) were increased, while dCDP (FC = 0.72) and dUMP (FC = 0.77) were significantly decreased (Supplementary Table S1, Figure S4). The selective depletion of deoxynucleotides (dCDP, dUMP, and the trending decrease in dCMP and dTDP) is consistent with mitochondrial dysfunction and may reflect impaired ribonucleotide reductase activity or increased consumption during DNA damage responses.

In the 600 nm low-dose group, deoxyadenosine monophosphate (FC = 1.23) was increased while hypoxanthine (FC = 0.82) was decreased (Supplementary Table S2). In the 600 nm high-dose group, a strikingly different pattern emerged, as multiple nucleotide triphosphates and diphosphates accumulated, including ATP (FC = 1.68), CTP (FC = 2.32), dGTP (FC = 1.68), dCTP (FC = 1.58), ITP (FC = 1.50), CDP (FC = 1.42), ADP (FC = 1.23), dGDP (FC = 1.23), UDP (FC = 1.30), uracil (FC = 1.50), cyclic AMP (FC = 1.23), and IDP (FC = 1.22), while inosine (FC = 0.61), hypoxanthine (FC = 0.73), guanine (FC = 0.63), and 3′-AMP (FC = 0.80) were decreased (Supplementary Table S2, Figure S4). The broad accumulation of nucleotide triphosphates and other related intermediates in the 600 nm group is consistent with the activation of nucleotide metabolic flux, potentially supporting the energetic and biosynthetic demands of inflammatory signaling and transcriptional reprogramming. The concurrent depletion of amino acids (observed in both LC-MS and GC-MS) and accumulation of oxidized glutathione (FC = 1.44) further suggest a cellular state of heightened oxidative and metabolic stress in the 600 nm high-dose group.

## 4. Discussion

This study demonstrates that MSN-induced toxicity in A549 cells is strongly influenced by particle size. By combining ultrastructural observation with metabolomic and molecular assays, we show that 80 nm and 600 nm MSNs do not merely differ in the magnitude of toxicity; instead, they induce qualitatively different patterns of subcellular injury and metabolic reprogramming.

A key finding of this study is that 80 nm MSNs were closely associated with mitochondrial damage. TEM revealed mitochondria that displayed swelling, cristae disruption and mitophagy-like changes. We noted that 80 nm particles were observed within membrane-bound vesicles yet were associated with mitochondrial damage—an apparent paradox that may be explained by endosomal/lysosomal escape and subsequent cytosolic release of nanoparticles or silica ions, a phenomenon documented for silica-based nanomaterials [20,25,26]. This provides a plausible mechanistic link between vesicular uptake and mitochondrial exposure without requiring direct TEM visualization of intramitochondrial particles. Consistent with this ultrastructural injury, metabolomic analysis revealed perturbation of the TCA cycle, with significant decreases in fumarate and malate, accompanied by the accumulation of early TCA intermediates citrate and oxaloacetate. This pattern of early TCA cycle metabolite accumulation with depletion of distal intermediates is consistent with impaired mitochondrial oxidative metabolism and compensatory enhancement of glycolytic flux [27,28]. Similar shifts toward glycolysis have been described in cells undergoing mitochondrial stress, oxidative injury or inflammatory activation [17,29,30]. In this context, the increased glyceraldehyde-3-phosphate and pyruvate observed after 80 nm MSN exposure may represent an adaptive response to preserve ATP production when mitochondrial metabolism becomes inefficient.

By contrast, 600 nm MSNs appeared to act mainly through disturbance of endocytic vesicles and intracellular membrane organization. TEM images showed vesicle membrane disruption and compression of intracellular structures rather than severe mitochondrial cristae loss. This form of physical stress may promote ROS generation and Ca^2+^ dysregulation [31,32,33]. The marked increase in HIF1A mRNA, together with strong induction of IL1B, IL6, and TNFA, supports the presence of a robust inflammatory transcriptional response in the 600 nm group. HIF-1α is not only a hypoxia-responsive transcription factor but also an important regulator of inflammatory metabolism and glycolytic adaptation [34,35]. Its transcriptional upregulation may therefore connect vesicular stress with metabolic remodeling and cytokine expression [13,36].

The metabolomic profiles further support a size-specific mechanism of toxicity. The 80 nm MSNs reduced several deoxynucleotide-related metabolites, including dCDP and dCMP, particularly at the higher dose. Such depletion may reflect impaired nucleotide synthesis or increased consumption during DNA repair and stress responses [37,38]. In contrast, 600 nm MSNs increased multiple nucleotide triphosphates, including ATP, CTP, dGTP, dCTP, CDP, ADP, and dGDP, accompanied by the depletion of amino acids—aspartic acid, glutamine, isoleucine, methionine, tyrosine, and tryptophan. These findings challenge the simplified view that nanomaterial toxicity uniformly suppresses cellular metabolism [23]. Instead, MSN exposure can trigger distinct metabolic states depending on particle size and subcellular localization.

Our results are consistent with earlier reports showing that silica nanoparticle toxicity is influenced by size, dose, and cell type [12,17,23]. Previous studies have emphasized ROS generation, membrane damage, and mitochondrial dysfunction as important mechanisms of silica nanoparticle toxicity [17,28,39]. The present study extends these findings by linking organelle-level injuries to specific metabolic pathway changes. Both LC-MS and GC-MS platforms provided convergent evidence for the size-dependent metabolic phenotypes. The proposed model can be summarized as follows: under the size conditions tested, 80 nm MSNs were predominantly associated with mitochondria injury, characterized by TCA cycle blockade (early intermediate accumulation with distal intermediate depletion), glycolytic compensation, and reduced deoxynucleotide synthesis; 600 nm MSNs primarily damaged endocytic vesicles and intracellular membranes, as characterized by broad nucleotide triphosphate accumulation, amino acid depletion, elevated oxidized glutathione, and amplified inflammatory signaling, including increases in IL1B expression and HIF1A transcriptional upregulation.

Several limitations of this study should be acknowledged. First, only two particle sizes (80 nm and 600 nm) were tested, representing a binary size contrast rather than a continuous dose–response gradient; future studies incorporating a broader range of particle sizes (e.g., 30, 100, 200, and 400 nm) would be required to determine whether the observed mitochondrial-versus-vesicular differences represent a true size threshold or a more gradual transition. Second, the TEM analysis is qualitative and descriptive; quantitative stereological analysis was not performed. Immunogold TEM or energy-dispersive X-ray spectroscopy (EDS) would be required to definitively confirm nanoparticle localization within specific organelles, and the current TEM evidence provides morphological association rather than direct co-localization [40,41]. Third, key mechanistic inferences rely on correlative evidence: metabolite abundance changes and mRNA levels provide convergent support but do not constitute causal proof. Direct mitochondrial functional validation (e.g., Seahorse oxygen consumption rate, mitochondrial membrane potential by JC-1/TMRM, ATP quantification, mitochondrial ROS) was not performed [42,43]. Fourth, the A549 cell line, while widely used, is a cancer-derived cell line and may not fully recapitulate the responses of normal lung epithelial cells; primary or immortalized normal lung epithelial models would provide complementary evidence. Finally, a nanoparticle-only MTT interference control (cell-free) was not included, and future studies should incorporate this control to definitively exclude optical or chemical interference by MSNs. Future studies should include a positive cytotoxicity control (e.g., Triton X-100 or staurosporine) to benchmark the MTT assay’s dynamic range. These limitations notwithstanding, the integrated multi-platform approach employed here provides a useful foundation for future mechanistic studies.

## 5. Conclusions

This study reveals that the cytotoxic effects of MSNs in A549 cells depend strongly on particle size and associated subcellular distribution. The 80 nm MSNs were predominantly associated with mitochondrial injury, TCA cycle disruption, and compensatory glycolytic metabolism, whereas 600 nm MSNs primarily disturbed endocytic vesicles, accompanied by HIF1A transcriptional upregulation, inflammatory signaling, and nucleotide metabolic remodeling. These findings establish a correlative link between MSN size, organelle damage, metabolic reprogramming, and inflammatory response. The results highlight particle size as a critical design parameter for safer MSN-based nanomaterials and provide candidate metabolic and inflammatory markers for nanotoxicological evaluation. These findings warrant functional validation by targeted mitochondrial respiration and membrane potential assays in future studies.

## Data Availability

The data presented in this study are contained within the article and Supplementary Materials.

## Supplementary Materials

The following supporting information can be downloaded at: https://www.mdpi.com/article/10.3390/metabo16080559/s1, Figure S1: Characterization of mesoporous silica nanoparticles: (A) 80 nm and (B) 600 nm; Figure S2: Cell viability. A549 cells exposed to MSNs with different sizes at concentrations ranging from 0 to 1000 μg/mL for 24 h using MTT assays. Data are expressed as the mean ± SD, *, *p* < 0.05, **, *p* < 0.01 and ***, and *p* < 0.001 compared with each 0 μg/mL group; Figure S3: Size effects of MSNs on energy metabolism in A549 cells. Heatmap of energy metabolites; Figure S4: Size effects of MSNs on purine and pyrimidine metabolism in A549 cells. Heatmap of purine and pyrimidine metabolites; Table S1: Significantly altered metabolites in A549 cells treated with 80 nm MSNs, identified by LC-MS (with FDR-adjusted q-values); Table S2: Significantly altered metabolites in A549 cells treated with 600 nm MSNs, identified by LC-MS (with FDR-adjusted q-values); Table S3: Significantly altered metabolites in A549 cells treated with 80 nm MSNs, identified by GC-MS (with FDR-adjusted q-values); Table S4: Significantly altered metabolites in A549 cells treated with 600 nm MSNs, identified by GC-MS (with FDR-adjusted q-values); Table S5: Primers used for gene expression analysis; Table S6: Raw Ct values for ACTB and GAPDH across all experimental groups.
