# Supplementary material for: Size-Dependent Metabolic Reprogramming in A549 Cells Induced by Mesoporous Silica Nanoparticles: Insights from Subcellular Targeting
Source: Metabolites. 2026 Aug 7;16(8):559. doi: 10.3390/metabo16080559 (PMC13515717; doi:10.3390/metabo16080559)
Supplement: Supplementary file 1 [file metabolites-16-00559-s001.zip › supplementary data.pdf]

Size-Dependent Metabolic Reprogramming in A549 Cells Induced by Mesoporous Silica Nanoparticles: Insights from Subcellular Targeting

Jing Li[a,*] and Hui Xu[b,c]

[a] Simulation and Control of Complex Ecosystem in Dabie Mountains of Anhui Higher Education Institutes, Key Laboratory of Modeling, School of Physics and Astronomy, Anqing Normal University, Anqing 246133, China.

[b] Key Laboratory of Drug Metabolism and Pharmacokinetics, State Key Laboratory of Natural Medicines, China Pharmaceutical University, Nanjing 210009, China.

[c] Lab of Nano-Biology Technology, Institute of Super-Microstructure and Ultrafast Process in Advanced Materials, School of Physics , Central South University, Changsha 410083, China.

* Correspondence: id_lijing@foxmail.com (Jing Li)

Table S5. Primers used for gene expression analysis

Primers	Sequence (5'-3')	Strain
TNFA	Forward CAGCCTCTTCTCCTTCCTGA<br>Reverse GGAAGACCCCTCCCAGATAGA	Human
IL6	Forward AAGCCAGAGCTGTGCAGATGAGTA<br>Reverse TGTCCTGCAGCCACTGGTTC	Human
Il1B	Forward TGTACCCAGAGAGTCCTGTGC<br>Reverse TCCTCTTAGCACTACCCTAAGGC	Human
HIF-1A	Forward AGACAAAGTTCACCTGAGCC<br>Reverse GGGAGCTAACATCTCCAAGTCT	Human
β-Actin	Forward AAACTGGAACGGTGAAGGTG<br>Reverse AGAGAAGTGGGGTGGCTTTT	Human
GAPDH	Forward AGAAGGCTGGGGCTCATTTG<br>Reverse AGGGGCCATCCACAGTCTTC	Human

Table S6. Raw Ct values for ACTB and GAPDH across all experimental groups

Experiment	Group	ACTB Ct	GAPDH Ct	ΔCt
80 nm	Control	13.48 ± 0.10	12.84 ± 0.26	0.64
80 nm	50 μg/mL	13.02 ± 0.33	11.80 ± 0.41	1.22
80 nm	200 μg/mL	14.85 ± 0.75	13.06 ± 0.25	1.79
600 nm	Control	13.29 ± 0.21	12.49 ± 0.33	0.80
600 nm	50 μg/mL	13.11 ± 0.37	12.06 ± 0.41	1.05
600 nm	200 μg/mL	16.44 ± 0.73	15.75 ± 0.10	0.69

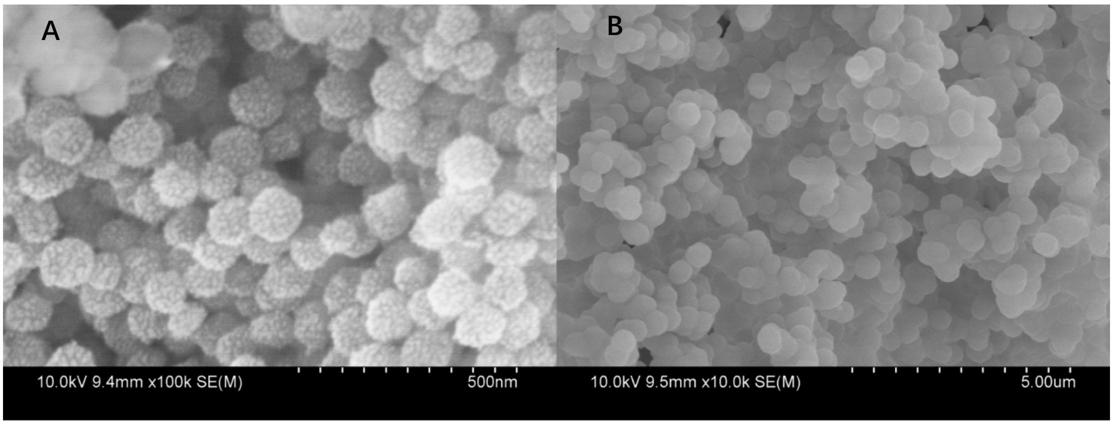

Fig. S1. characterization of mesoporous silica nanoparticles
(A) 80 nm, (B) 600 nm

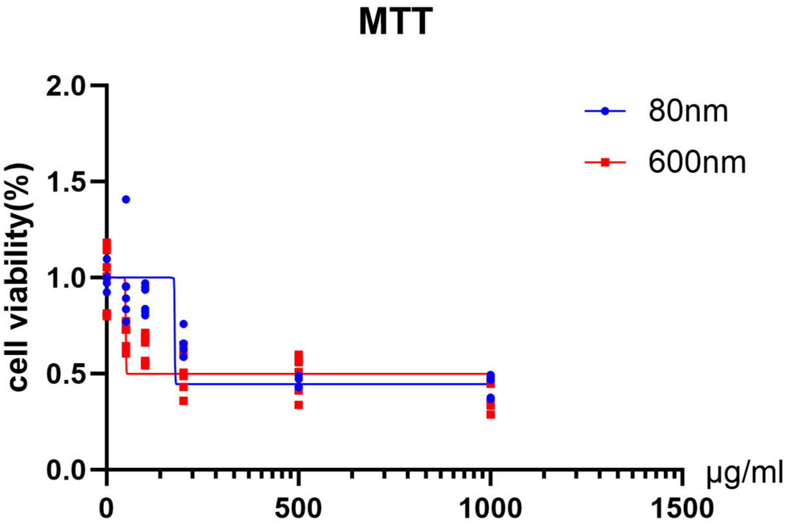

Fig. S2. Cell viability.
A549 cells exposed to MSN with different sizes at concentrations ranging from 0-1000 μg/mL for 24 h using MTT assays. The data are expressed as the mean ± SD, ∗, $p < 0.05$, ∗∗, $p < 0.01$ and ∗∗∗,$p < 0.001$ compared with each 0 μg/ml group.

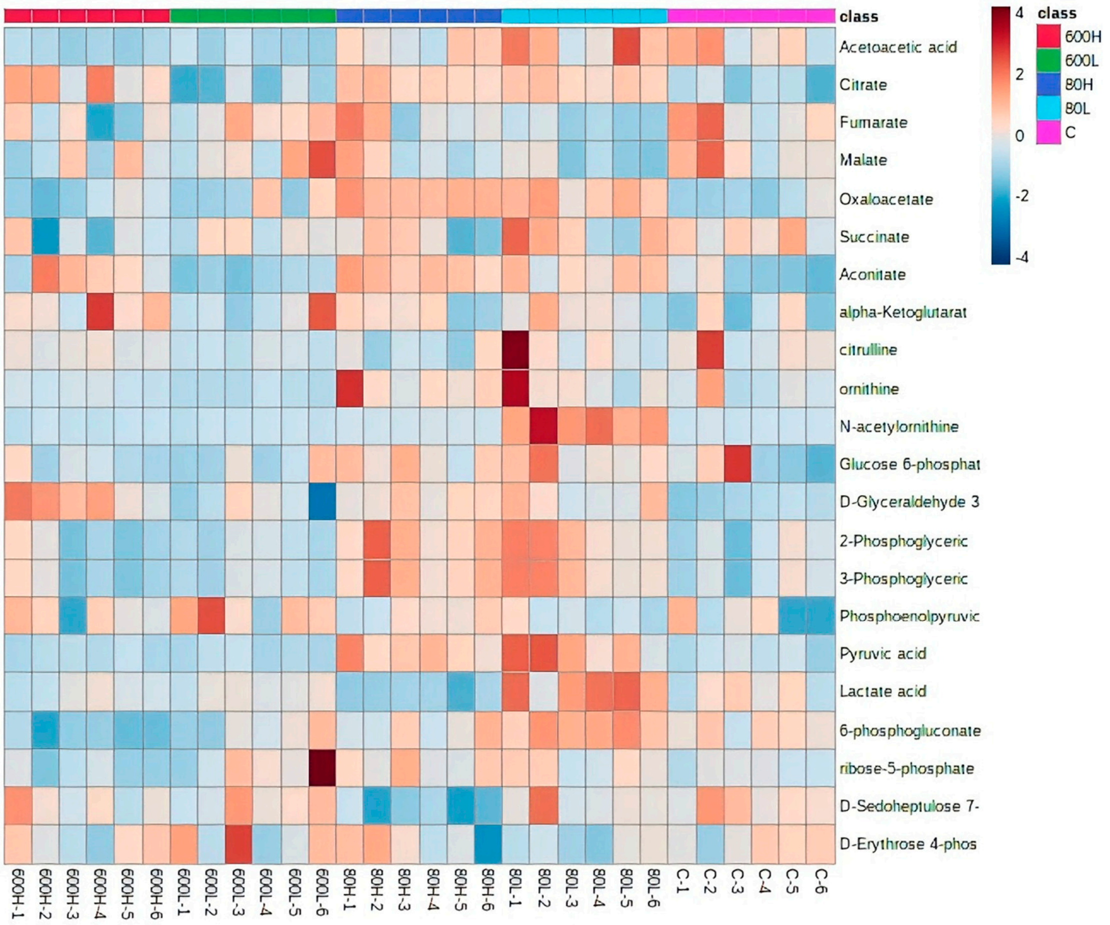

Figure S3. Size effects of MSNs on energy metabolism in A549 cells.
The heatmap of energy metabolites.

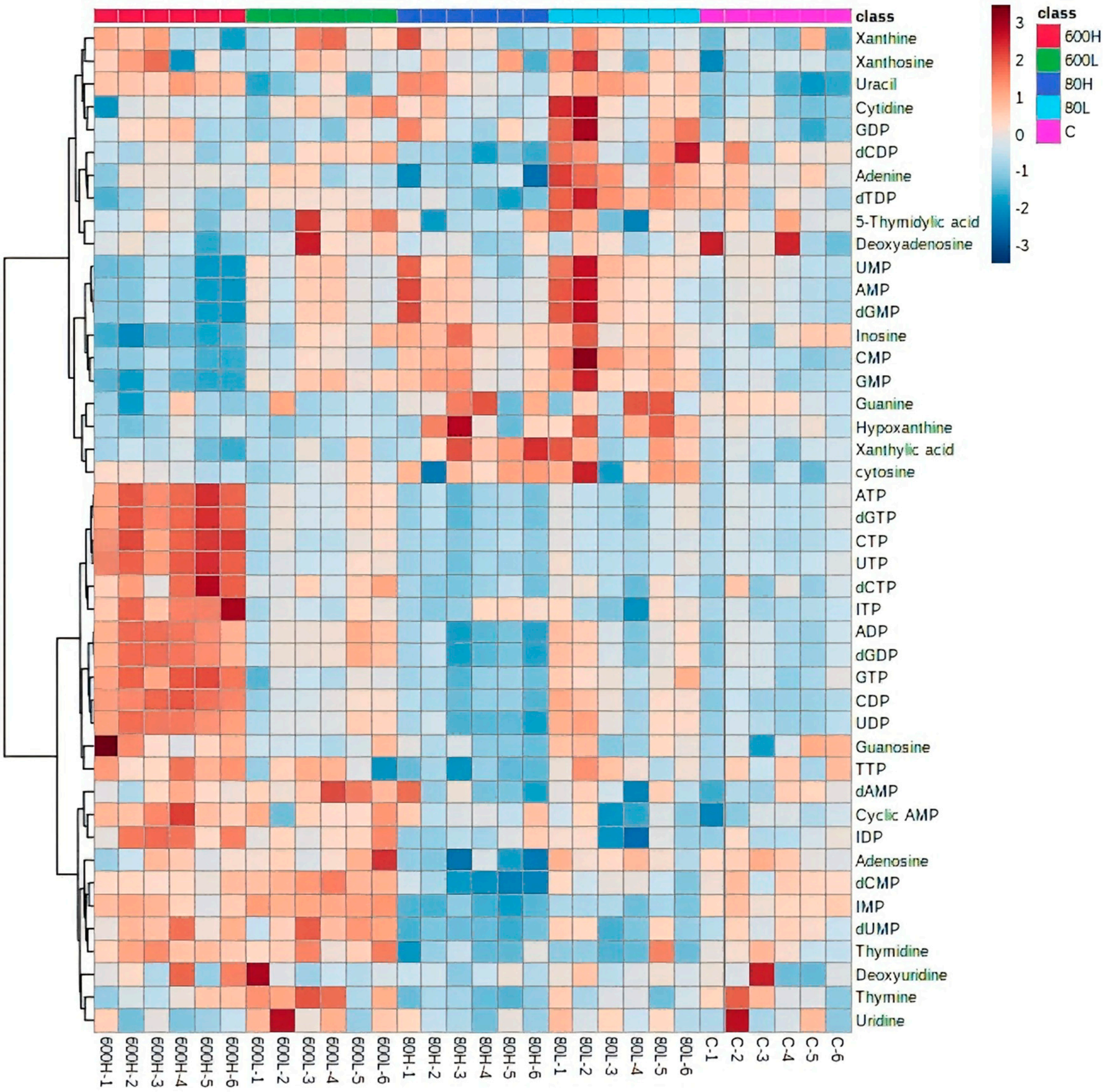

Figure S4. Size effects of MSNs on pyrimidine metabolism in A549 cells.
The heatmap of purine and pyrimidine metabolites.
